# Another perspective of strain selection based on functional traits: construction and evaluation of a key complex index for endangered species plantation

**DOI:** 10.3389/fpls.2025.1511693

**Published:** 2025-03-14

**Authors:** Jinshi Xu, Biao Han, Dan Liu, Jintao Pang, Huixin Guo, Xiang Zhou, Ping Zhang

**Affiliations:** ^1^ School of Life Sciences, Ludong University, Yantai, Shandong, China; ^2^ Shandong Provincial Center of Forest and Grass Germplasm Resources, Jinan, Shandong, China; ^3^ Kunyu Mountain Forest Farm, Yantai, Shandong, China

**Keywords:** *J. mandshurica*, measurable trait, strain optimization, management needs, insect resistance

## Abstract

**Introduction:**

Endangered species can achieve population growth through utilization. *Juglans mandshurica* is an endangered species, which can be used in gardens and street trees. To avoid population degradation caused by long-term nursery cultivation, we need to introduce high-quality wild sources of germplasm for hybridization. In the past, when the selection of strains was carried out, attention was often paid to the performance of different traits of each strain. The strains with advantages in many more traits were selected as the target. In this paper, we proposed that excellent strains should be selected based on the needs of managers.

**Methods:**

We constructed a complex index composed of insect resistance and growth amount, which was concerned by plantation managers, for the selection of excellent strains. Its availability was confirmed as well. We cultivated 16 wild-sourced *J. mandshurica* strains in a homogeneous garden and carried out experiments for 3 years. We measured 28 functional traits. Through collinearity diagnostics, 15 functional traits in 4 dimensions (morphology, leaf economy, stoichiometry and reproduction) were selected for analysis and construction of complex index. The influence of environmental factors on traits was excluded by comparing the trait matrix calculated based on Euclidean distance with the geographical distance matrix.

**Results:**

Excellent strains (No. 15 from Dazeshan) selected based on the key complex index may not be outstanding in each trait, but have a more balanced performance among the trade-offs of trait combinations. We also explored the visualization of this key complex index by correlating with leaf carbon content (its ecologically relevant trait), so as to realize rapid and early selection of *J. mandshurica* strains by using LCC (an easily measurable trait).

**Discussion:**

To construct key complex index, appropriate functional traits should be selected according to the needs of managers or different species. The measurable traits with clear ecological links with complex index should be selected as "agents" to realize visualization of complex index.

## 1 Introduction

Another way to preserve endangered species is to use them. Many endangered tree species have horticultural value and form large artificial populations in cities. However, the long-term breeding of the species in the nursery may led to the degradation of the fine traits of the endangered species (Yang et al., 2021). For example, long-term inbred or clonal reproduction reduces the genetic diversity of species and causes serious homogeneity of traits, which leads to serious diseases in some nurseries. Due to the limited germplasm of endangered species, it is more likely to use closely related plants as parents, or simply to reproduce asexually. Regular collection of germplasm from natural populations of endangered species in the wild and their introduction into nurseries for hybridization are therefore necessary to maintain the genetic and functional diversity of endangered species’ artificial populations. Researchers select excellent strains from germplasm through functional traits. For example, morphological traits can indicate how efficiently a plant uses light or nutrients; The stoichiometric traits can indicate the photosynthetic capacity and disease resistance of plants. Economic traits can explore plant trade-offs with life-history strategies (Laughlin, 2014). However, the traits of an endangered population for landscaping use should serve its specific use. As street trees, for example, managers often consider their swiftness of growth and ability to resist predators. Therefore, when introducing wild germplasm, it is still necessary to assess whether the “key traits” of concern meet the needs of managers.

*Juglans mandshurica* is a deciduous broad-leaved tree species in northern China. Previous studies have shown that fructus and leaves of *J. mandshurica* have anticancer chemical components (Guo et al., 2015; Luan et al., 2021). Meanwhile, the woody material of *J. mandshurica* is excellent, which has high utilization value (Wang et al., 2016). Due to human logging and habitat loss, *J. mandshurica* is rarely seen in some distribution areas (Zhang et al., 2022). It has been identified the endangered plants in China (State Environmental Protection Bureau & Institute of Botany, Chinese Academy of Sciences, 1987; State Forestry Bureau, 1992). Recently, there has been some expansion of the wild population of *J. mandshurica*, and its genetic diversity is relatively high (Wu, 2013; Wang et al., 2023). Therefore, it is feasible to supplement plantation provenance from the wild, and it is worth studying.

*J. mandshurica* tree has beautiful shape and can be used as landscape trees and street trees. In the conservation and utilization of *J. mandshurica*, it is necessary to introduce wild germplasm resources regularly in the nursery and screen the concerned traits of wild germplasm resources.

The functional trait of plant can be associated or orthogonal (Poorter et al., 2014). In general, traits controlled by a single gene are orthogonal to each other (such as those in Mendel’s pea experiment, Cosmulescu et al., 2018). However, many studies have shown that traits that are relatively independent genetically can be phenotypically related to each other (Ipek et al., 2019). The selection of key trait combinations and the correlation analysis between measurable traits and key trait combinations can provide reference for the breeding of excellent traits. Researchers have proposed that the study of community based on functional traits needs to cover at least four dimensions (Laughlin and Messier, 2015). Therefore, the selection of excellent strains at the population level should also refer to the performance of traits in multiple dimensions. For example, we can pay attention to leaf economic traits, morphological traits, stoichiometric traits, but also take into account the survival and budding rates of the strains. Leaf economic traits represent plant growth strategies (He et al., 2006). For example, species that tend to grow fast tend to have smaller specific leaf areas and shorter leaf lifespans (Wilson et al., 1999). Morphological traits can reflect the ability of species to accumulate photosynthetic products, and have a positive correlation with biomass (di Cosmo et al., 2016; Yin et al., 2019). Leaf stoichiometric traits can reflect the photosynthetic capacity of species and the ability to resist pests and diseases (Chai et al., 2015; Xu et al., 2018). Germination rate and survival rate represent the intrinsic reproductive properties of species, and also reflect the trade-off of the growth-reproduction relationship (Shipley et al., 2006; Enquist et al., 2007).

In this study, we collected seeds of different stable mature wild populations for germination experiment, and further planted seedlings of different strains in a homogeneous garden for 3 years (because seedlings were influenced by micro-habitat significantly, Li et al., 2018). We collected multi-dimensional traits from each strain and constructed key complex index that managers focused on to evaluate the quality of each strain as an excellent germplasm candidate. Meanwhile, we analyzed its availability, and tried to find measurable traits associated with this key trait, as a visual agent to early selection of high-quality strains. Through the above experiments, this study tried to reveal: 1. Whether the key index based on the needs of managers provides a new perspective in selection of excellent strains 2. Whether rapid selection of excellent strains for a particular tree species can be achieved through easily measurable traits.

## 2 Materials and methods

### 2.1 Collection of germplasm

In September 2019, fruits were collected from a typical and stable population of *J. mandshurica* in Shandong, China, which is located on the southern margin of its distribution and has high genetic diversity (Wang et al., 2023). The specific sampling sites are shown in **Table 1**. Sample sites are the locations of the trees from which we collected the seeds. We selected a single tree of *J. mandshurica* with good growth status in the central area of each population. We collected its naturally mature and pleins fruits and carried out fruit treatment. Since the seeds of *J. mandshurica* are recalcitrant seeds and cannot withstand storage, the treated fruits would be subjected to germination experiments as soon as possible. We collected 1253 seeds from 16 trees belonging different provenance sites for germination experiments.

**Table 1:** Provenance sites and traits of germplasm stock trees of *J. mandshurica*.

Germplasm code	Serial number	Site	Geographical coordinates (°)		Elevation (m)	Stock tree basal diameter (cm)	Stock tree DBH (cm)	Stock tree height (m)	Stock tree crown breadth (m)
1	MSM1	Mengshan Mountian, Pingyi	35.5399N	117.8373E	520	28.0	26.0	8.4	7.4×6.9
3	MSM2	Mengshan Mountian, Pingyi	35.5421N	117.8428E	559	30.2	19.8	13.7	10.4×11.5
2	MSM3	Mengshan Mountian, Pingyi	35.5421N	117.8428E	559	21.1	18.3	12.6	6.6×4.9
4	MSM4	Mengshan Mountian, Pingyi	35.5386N	117.8358E	429	35.5	20.5	11.3	9.0×8.6
5	MSM5	Mengshan Mountian, Pingyi	35.5427N	117.8446E	587	30.5	21.2	10.4	8.1×7.6
6	MSM6	Mengshan Mountian, Pingyi	35.5427N	117.8446E	587	29.4	23.9	9.1	9.4×9.7
7	MSM7	Mengshan Mountian, Pingyi	35.5530N	117.8503E	1032	34.2	27.9	8.4	8.5×8.4
8	MSM8	Mengshan Mountain, Mengyin	35.557841N	117.927182E	555	23.7	17.1	7.7	10.4×11.3
9	TSM1	Culai Mountain, Tai’an	36.0422N	117.3151E	778	26.6	22.7	10.6	12.7×11.9
10	KYM1	Kunyu Mountain, Muping	37.2692N	121.7288E	572	22.1	15.6	8.5	4.0×3.2
11	KYM2	Kunyu Mountain, Muping	37.5384N	121.7721E	201	24.4	15.5	9.3	5.5×5.0
12	KYM3	Kunyu Mountain, Muping	37.1604N	121.4303E	233	25.1	17.6	9.9	3.5×3.0
13	KYM4	Kunyu Mountain, Muping	37.2640N	121.7728E	480	25.5	18.9	10.5	6.8×5.7
14	KYM5	Kunyu Mountain, Muping	37.2617N	121.7750E	535	20.6	16.8	9.8	8.5×9.7
15	DZM1	Daze Mountain, Pingdu	36.9880N	120.0489E	256	22.3	19.8	10.2	10.4×9.7
16	DZM2	Daze Mountain, Pingdu	36.9880N	120.0388E	359	23.7	20.5	10.0	9.7×10.7

### 2.2 Homogeneous garden experiment

The collected seeds were simultaneously subjected to germination experiment, and the germination conditions remained the same. The final germination rate (GR) of each strain was recorded. After germination, the seedlings were transferred to the greenhouse for cultivation. In 2020, the final surviving seedlings were transplanted into the field. The selected field is located in Kunyu Mountain Forest Farm (37.3125°N, 121.7501°E). The field has been prepared, and the soil, water and heat conditions of the whole transplanting field are consistent, which would minimize the influence of environmental factors on strains traits. The number of seedlings which we transplanted was 490. The number of each strain seedling was at least 30. Strain seedlings from the same stock tree were planted together, and the seedlings were 4m apart from each other to avoid errors caused by competition for water, light and soil nutrients. After transplanting, the number of individuals who died was recorded monthly. The final survival rate (SR) of each strain was recorded during the third growing season of *J. mandshurica* seedlings in July 2022. A total of 450 seedlings were still survival at that time. In order to explore the differences in traits of different strains, 10 seedlings were randomly selected as experimental objects in different strains at the same time, and the number of each seedling was recorded. A total of 160 seedlings were analyzed and sampled.

**Figure 1:**
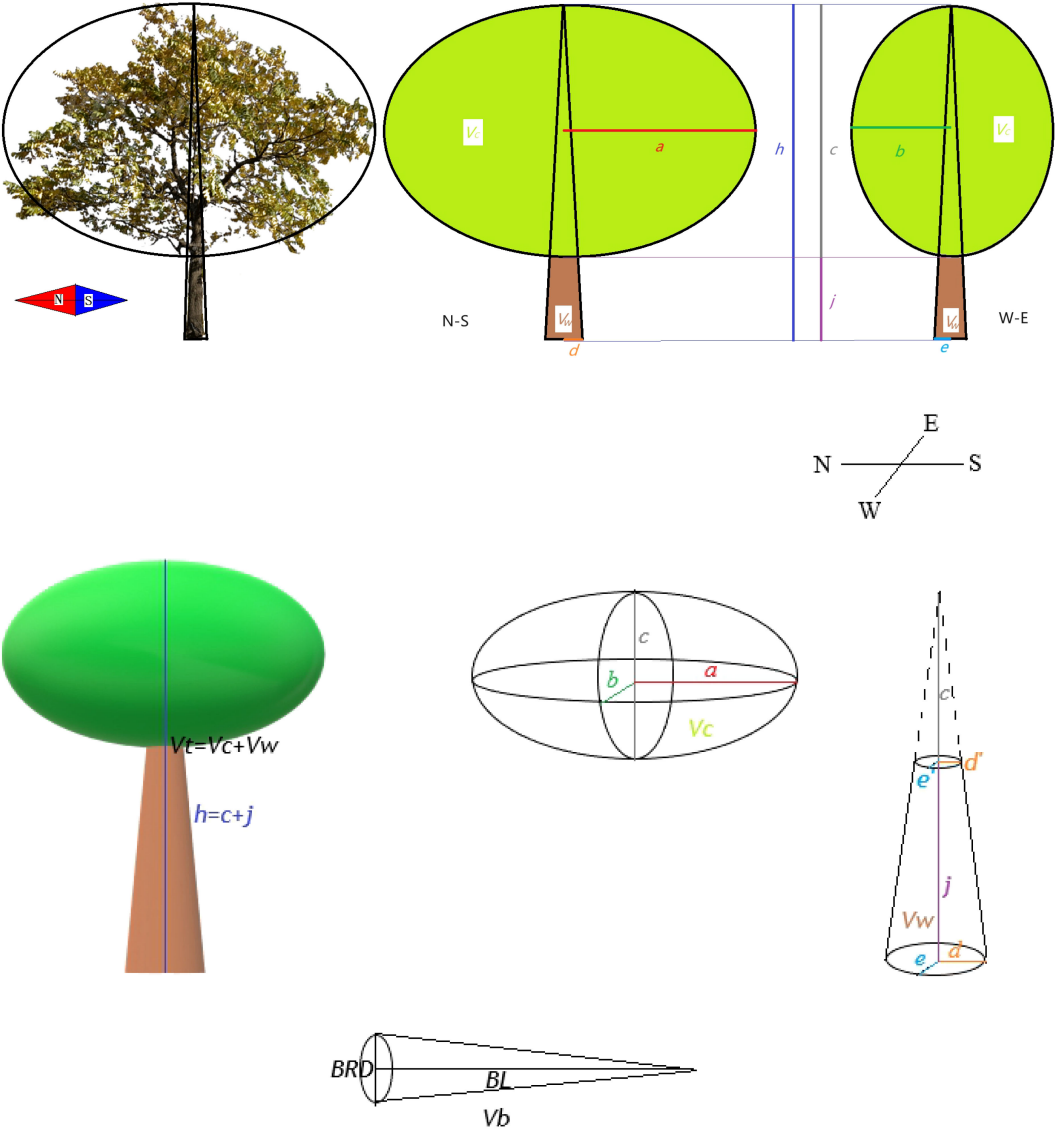
Example for measuring and calculating total tree volume (Vt) and annual branch volume (Vb). N means north, S means south, W means west and E means east. *Vw* is the trunk part volume while *Vc* is crown part volume. *a* is 1/2 major axis of crown breadth, *b* is 1/2 minor axis of crown breadth. *c* is crown length. *h* is plant height. *d* and *e* are 1/2 basal diameter. *j* is height below the branch. *BRD* is annual branch middle diameter. *BL* is annual branch length.

The traits involved in the analysis are shown in **Table 2**. In order to achieve the calculation of the above traits, the plant morphological indexes that need to be measured in the field experiment include crown width (2a from north to south, 2b from east to west, see **Figure 1**), base diameter (2d from north to south, 2e from east to west, see **Figure 1**), plant height (h), height below the branch (j), annual branch length (BL), and annual branch middle diameter (BRD). Each sample of annual branches measured five times, including four directions and one branch in the middle. If there are less than 5 annual branches, the actual number of branches were measured. At the same time, the lenticel number (many - medium - few) and the lenticel size (large - medium - small) of the seedlings were qualitatively recorded. The height of the tree is measured with a height recorder, and other length indicators are measured with meter ruler, DBH ruler and vernier caliper.

**Table 2:** Functional traits of strains of *J. mandshurica*.

Trait dimensions	Functional trait	Unit	Original measurable trait	Unit	Trait effect of plant
morphological traits	total tree volume (Vt)	m^3^	height (h)	m	productivity
			crown breadth (2a, 2b)	m	
			basal diameter (2d, 2e)	m	
			height below the branch (j)	m	
	leaflet number (LN)	slice	leaflet number (LN)	slice	Phylogenetic characteristics
	annual branch volume (Vb)	cm^3^	annual branch length (BL)	cm	The ability of plants to acquire resources
			annual branch middle diameter (BRD)	cm	
	relative lenticel area (RLA)	1	relative lenticel number (RLN)	1	Disease resistance
			relative lenticel size (RLS)	1	
	leaf length (LL)	cm	leaf length (LL)	cm	Phylogenetic characteristics
	leaf width (LW)	cm	leaf width (LW)	cm	
	uppermost leaf length (ULL)	cm	uppermost leaf length (ULL)	cm	
	uppermost leaf width (ULW)	cm	uppermost leaf width (ULW)	cm	
	lateral leaf length (LLL)	cm	lateral leaf length (LLL)	cm	
	lateral leaf width (LLW)	cm	lateral leaf width (LLW)	cm	
	petiole length (PL)	cm	petiole length (PL)	cm	
leaf economic traits	leaf water content (LWC)	%	leaf fresh mass (LFM) leaf area (LA) leaf dry mass (LDM)	g cm^2^ g	Life history strategies
	leaf area (LA)	cm^2^			Light utilization capacity
	leaf dry matter content (LDMC)	‰			Life history strategies
	specific leaf area (SLA)	cm^2^/g			Light utilization capacity
stoichiometric traits	soluble sugar content (SSC)	mg/g	soluble sugar content (SSC)	mg/g	Carbon sequestration capacity
	leaf nitrogen content (LNC)	%	leaf nitrogen content (LNC)	%	Photosynthetic capacity
	leaf nitrogen content (LCC)	%	leaf nitrogen content (LCC)	%	Carbon sequestration capacity
	borer-resistance (BR)	1	polyphenol content (PC)	mg/g	Disease resistance
			tainnin content (TC)	mg/g	
reproductive traits	germination rate (GR)	%	germination rate (GR)	%	survivability
	survival rate (SR)	%	survival rate (SR)	%	

Leaf economic traits mainly focus on leaf area (LA), leaf dry matter content (LDMC), leaf water content (LWC), specific leaf area (SLA) was classical indexes (Bin et al., 2022). At the same time, leaf morphological traits such as leaf length (LL), leaf width (LW) and petiole length (PL) were also measured. We also recorded uppermost leaf length (ULL), uppermost leaf width (ULW), lateral leaf length (LLL), lateral leaf width (LLW), and leaflet number (LN), as the leaves of *J. mandshurica* are odd-pinnate compound leaves. The stoichiometric characteristics of leaves we selected were carbon content (LCC), nitrogen content (LNC), polyphenol content (PC), tannin content (TC) and soluble sugar content (SSC). In order to analyze the above traits, 5 complete compound leaves with maturity and development in all directions were selected as leaf samples for each seedling as the experimental object during field measurement. The leaf samples were stored in wet specimen paper and brought back to the laboratory.

### 2.3 Laboratory experiment

Immediately after the 5 leaves of each seedling were brought back to the laboratory, they were weighed fresh mass. Their leaf profiles were scanned as well. Based on the standard method, the leaf area (LA), leaf dry matter content (LDMC), leaf water content (LWC) and specific leaf area (SLA) of each selected seedling were calculated (Perez-Harguindeguy et al., 2013). Leaf scanning was performed using the EPSON V300 scanner. The LA and LN were measured and counted using ImageJ Pro Plus software. After that, the rest morphological traits of the leaves were measured with a ruler. The leaves will be loaded into a paper bag and dried at 70°C for 48h to constant weight to calculate the dry matter content and water content of the leaves. The dried leaves were crushed, screened with 100 mesh, and their stoichiometric traits were measured by standard method. The stoichiometric indices of each individual are the average values measured for the 5 leaves.

### 2.4 Construction of complex index

A single plant morphological trait may have no physiological or ecological significance. In order to make the above traits clearly point to function and reduce the number of indexes involved in analysis, we pretreated the plant morphological indexes. We constructed two indexes of total tree volume (Vt) and annual branch volume (Vb).

The trees in **Figure 1** are the real pictures of the young trees of *J. mandshurica*. For garden managers, they pay more attention to the overall volume of trees, and the larger the volume, the better the landscape effect or the greater productivity (rapid growth). Vt can represent the size of the spatial niche (such as light, nutrients, water, etc.) occupied by the plant (Stewart et al., 2005), and indirectly reflect the biomass accumulation of the plant during the same time (because the wood density and branching pattern of the same species are conservative, di Cosmo et al., 2016). Therefore, we developed Vt as an indicator to estimate the volume of young trees. For a tree like the *J. mandshurica*, the spatial volume of the tree is roughly equal to the volume of the crown (Vc) plus the volume of the trunk (Vw) (**Figure 1**). Due to the ellipsoidal crown of the *J. mandshurica*, the rotational volume of the crown can be approximated to the ellipsoidal volume; referring to Li (2009) for the correction formula of ellipsoidal crown volume, Vc was calculated based on plant morphology index. The Vw can be regarded as the difference of the elliptic cone with height as plant height minus the elliptic cone with height as crown length (**Figure 1**).

Meanwhile, Vb represents the growth status of the annual branches in the current year, reflecting the functional response of the plants in a specific time period (Osnas et al., 2013). The annual branch is relatively straight and unbranched, and we can think of it as a uniformly cone. The volume of annual branches was characterized by the cone volume with BL as the height and BRD/2 as the base radius.

The calculation formula of Vt and Vb is as follows:


h=c+j, c=h−j

$$Vc=\frac{\pi c(a^{2}+b^{2})}{3}=\frac{\pi (h-j)(a^{2}+b^{2})}{3}$$

$$d'=\frac{cd}{h}=\frac{(h-j)d}{h},\;e'=\frac{ce}{h}=\frac{(h-j)e}{h}$$

$$Vw=\frac{\pi deh}{3}-\frac{\pi d'e'c}{3}=\frac{\pi deh}{3}-\frac{\pi {\left(h-j\right)}^{3}de}{3h^{2}}$$

Vt=Vc+Vw

$$=\frac{\pi (h-j)(a^{2}+b^{2})}{3}+\frac{\pi deh}{3}-\frac{\pi {\left(h-j\right)}^{3}de}{3h^{2}}$$

$$=\frac{\pi (h-j)(a^{2}+b^{2})+\pi deh}{3}-\frac{\pi {\left(h-j\right)}^{3}de}{3h^{2}}$$

(1) $$=\frac{\pi }{3}[(h-j)(a^{2}+b^{2})+deh-\frac{{\left(h-j\right)}^{3}de}{h^{2}}]$$

Equation 1 is calculated based on the ellipsoidal volume formula and the ellipsoidal table volume formula.


(2) $$Vb=\text{π}(BL){\left(BRD/2\right)}^{2}/3$$

Equation 2 is calculated based on the cone volume formula.

Single morphological traits who participate in construction of Vt and Vb would no longer participate in subsequent analysis to avoid data redundancy. In the subsequent analysis, Vt and Vb were used as functional morphological indexes.

For the lenticel traits, we assigned the qualitative traits of lenticel size, and number to 1 for small (few), 2 for medium and 3 for large (many). We multiplied the data on the relative lenticel size (RLS) with relative lenticel number (RLN) to obtain the relative lenticel area (RLA) for each individual. In the subsequent analysis, RLS and RLN were no longer involved in the analysis.

As for stoichiometric indicators, studies have shown that the polyphenol (PC) and tannin contents (TC) of leaves are related to their resistance to herbivorous species (Xu et al., 2022). We standardized the PC and TC of each individual under test using the extremum method, converting the data to [0.001, 1]. To avoid interference from the minimum (normalized to 0) in subsequent analyses, we set the normalized value of the minimum to 0.001. We use the product of standardized PC and TC as a newly constructed pest resistance index (BR). In subsequent analyses, PC and TC were no longer involved in the analysis.

In the application of artificial population of *J. mandshurica*, street trees and landscape trees are the main utilization ways. Managers in the application, often pay attention to the robustness of its growth and the ability to resist pests and insects, in order to save the cost of fertilization and deworming. Therefore, tree growth amount and insect resistance are the key traits to evaluate the use of *J. mandshurica* as a garden tree. We multiply standardized BR with standardized Vt to construct key complex index that are of concern to managers. Since the value of complex index is quite small, we log-transformed the value and use log-complex index as the characteristic of strain goodness of different *J. mandshurica* individuals.

### 2.5 Statistical analysis

**Figure 2:**
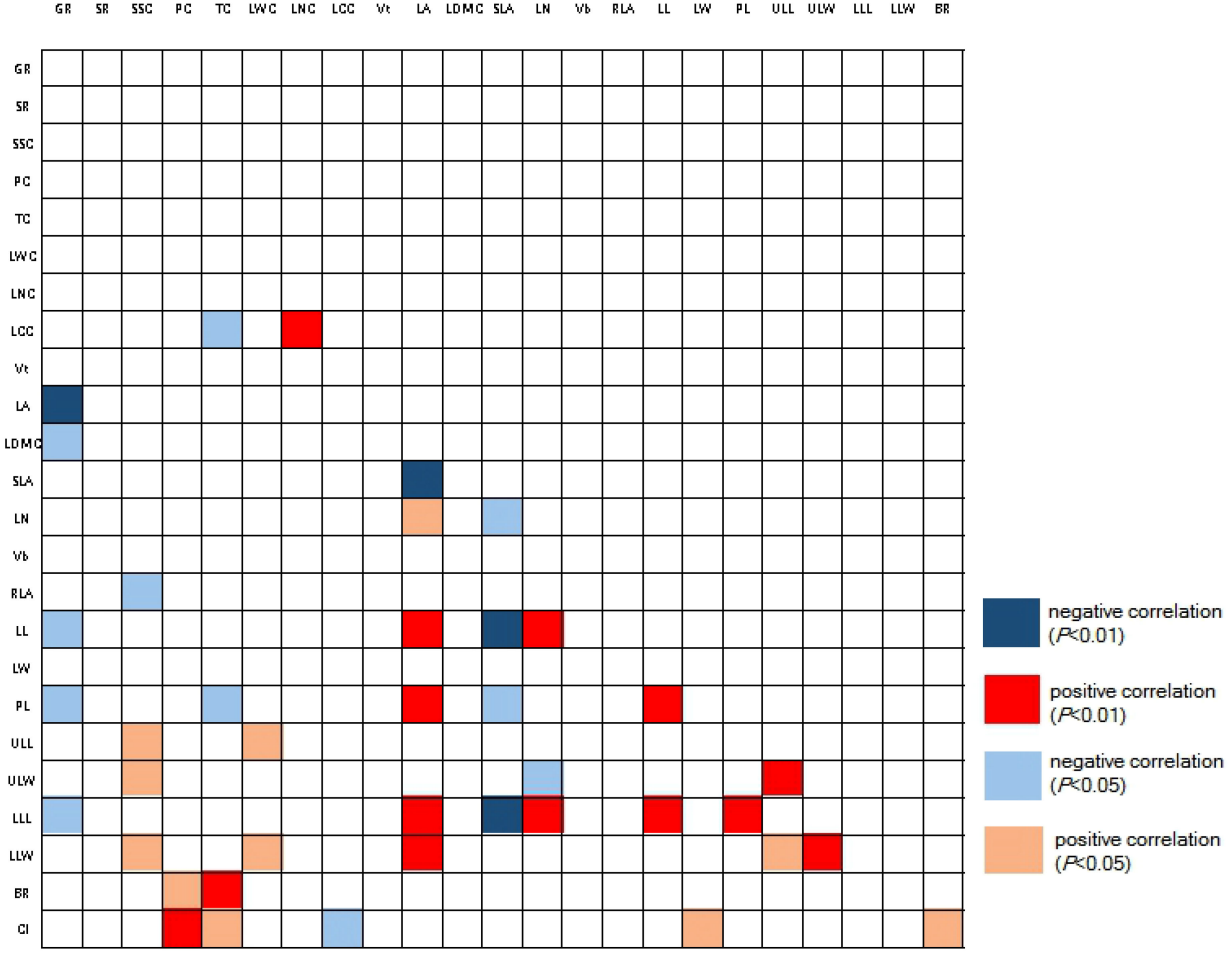
Pearson correlation of traits among strains. Red color refers to positive correlations with each other, while blue color refers to negative correlation. The abbreviation of traits see **Table 2**. CI, complex index.

Many traits have interrelated properties (**Figure 2**). In order to avoid the interference of multicollinearity in the subsequent analysis, we calculated the variance inflation factor (VIF) of the traits. Based on Akaike information criteria, traits with small VIF and relative independence were selected (see **Table 2**). Eventually participate in the analysis of the traits are, morphological traits: Vt, LN, Vb, RLA, LW, PL, ULL, LLW; Stoichiometric traits: LNC, LCC, BR; Leaf economic traits: LA, LDMC; Reproductive traits: GR, SR. The traits in the final analysis are easy to measure and have clear ecological significance. They are still distributed in four dimensions. The values of the above traits were calculated for each individual in each strain, and the mean value of functional traits and coefficient of variation (CV) were calculated. ANOVA was used to determine the difference degree of the above traits among the strains, and to label the traits that are of concern. When labeling, the strains with the largest and smallest mean trait values were labeled, and the strains with no significant difference from the maximum and minimum values were also labeled as candidate strain if their CV was less than 15 (or the minimum value of CV in all strains, indicating excellent trait stability within the strain). Among the candidate strain, those that had positive effects on the characteristics concerned by managers (fast growth, good insect resistance, strong photosynthetic capacity, etc.) were marked as +, and those that had negative effects on the traits concerned were marked as - (**Table 3**). We counted the number of advanced traits of each strain and compared them with the complex index we constructed. In the statistical process, we found that the overall CV of SR, LNC and LCC was very small (CV<10), indicating that these traits may be conserved traits. Therefore, the above three traits were not counted when the advantages of single functional traits were counted.

**Table 3:** Mean functional traits and complex index of each strain.

Traits	Strain mean trait value															
	1	2	3	4	5	6	7	8	9	10	11	12	13	14	15	16
GR	32.39	70	**3.11-**	53.54	3.57	33.33	55.26	73.17	7.69	14.39	**73.91+**	66.67	35.94	46.77	45.05	61.25
SR	**100+**	**100+**	**100+**	**100+**	**100+**	**100+**	**78.57-**	92.16	88	**100+**	88.71	**100+**	85.29	96.42	**100+**	85.71
BR	0.07 ± 0.05	0.02 ± 0.01	0.03 ± 0.04	0.02 ± 0.01	0.01 ± 0.01	0.02 ± 0.01	0.08 ± 0.08	0.07 ± 0.09	0.05 ± 0.02	0.10 ± 0.06	**0.20 ± 0.26+**	0.02 ± 0.02	0.05 ± 0.04	0.07 ± 0.05	0.09 ± 0.06	0.06 ± 0.01
LNC	**2.07 ± 0.18-**	**2.24 ± 0.14+**	**2.07 ± 0.23-**	**2.23 ± 0.21+**	**2.61 ± 0.20+**	2.15 ± 0.42	**2.44 ± 0.33+**	**2.42 ± 0.25+**	2.30 ± 0.48	**1.80 ± 0.23-**	**2.11 ± 0.19-**	2.18 ± 0.37	**2.54 ± 0.24+**	**1.97 ± 0.24-**	2.28 ± 0.41	2.44 ± 0.42
LCC	**43.48 ± 0.44-**	**44.91 ± 0.45+**	**44.21 ± 0.31-**	44.32 ± 0.46	**45.41 ± 0.47+**	**44.89 ± 0.35+**	**44.80 ± 0.99+**	**44.63 ± 0.57+**	**44.97 ± 0.48+**	**43.71 ± 0.55-**	44.46 ± 0.46	**44.07 ± 0.34-**	**44.70 ± 0.87+**	**44.01 ± 0.71-**	**44.21 ± 0.78-**	**45.11 ± 0.56+**
Vt	1.95 ± 1.19	1.57 ± 1.39	**4.14 ± 4.76+**	1.34 ± 0.54	1.83 ± 1.39	1.24 ± 0.38	1.33 ± 0.29	2.39 ± 1.54	1.64 ± 0.51	1.66 ± 0.34	**0.91 ± 0.20-**	1.70 ± 0.80	1.51 ± 0.72	2.19 ± 1.08	2.75 ± 1.20+	1.99 ± 1.17
LA	677.86 ± 230.61	601.19 ± 171.34	719.37 ± 210.46	730.55 ± 233.66	**875.46 ± 199.06+**	803.63 ± 209.15	723.91 ± 268.54	665.37 ± 200.36	693.41 ± 246.22	**841.57 ± 246.90+**	679.85 ± 179.57	**554.62 ± 216.22-**	653.41 ± 181.93	609.32 ± 222.07	553.4 ± 217.53	572.62 ± 168.28
LDMC	**687.91 ± 110.46-**	654.16 ± 126.51	656.13 ± 53.24	636.54 ± 107.01	668.46 ± 32.09	**645.02 ± 38.85+**	**634.03 ± 47.82+**	**629.07 ± 38.67+**	**636.64 ± 56.05+**	680.15 ± 197.48	**639.7 ± 55.2+**	**616.63 ± 50.08+**	655.09 ± 39.40	649.56 ± 42.00	645.03 ± 80.94	655.13 ± 36.45
LN	14.20 ± 1.79	14.60 ± 2.19	14.67 ± 2.14	15.90 ± 1.97	**17.06 ± 1.83+**	15.50 ± 1.46	15.00 ± 2.29	13.83 ± 1.70	14.70 ± 1.99	15.13 ± 3.00	14.97 ± 1.25	**11.70 ± 2.55-**	15.77 ± 2.37	15.43 ± 1.28	16.43 ± 2.57	14.13 ± 2.21
Vb	901.29 ± 566.58	597.41 ± 423.87	**1288.97 ± 255.64+**	771.89 ± 603.7	**1210.32 ± 237.25+**	612.29 ± 503.1	**1477.93 ± 1458.56+**	1547.83 ± 1446.33	1352.29 ± 1298.25	1591.43 ± 1287.69	**504.53 ± 333.28-**	646.84 ± 516.87	769.80 ± 552.48	1053.44 ± 998.25	1474.24 ± 1285.96	797.49 ± 636.47
RLA	1.8 ± 1.1	1.40 ± 0.44	1.40 ± 0.89	2.00 ± 1.00	**3.00 ± 0.00-**	2.20 ± 0.84	**3.00 ± 0.00-**	2.20 ± 0.84	1.60 ± 0.89	2.80 ± 0.45	**1.20 ± 0.45+**	2.60 ± 2.07	2.2 ± 1.09	4.2 ± 2.68	2.40 ± 0.89	**3.00 ± 0.00-**
LW	28.82 ± 5.50	28.02 ± 3.65	**29.95 ± 4.08+**	30.33 ± 6.32	**32.97 ± 3.85+**	29.60 ± 5.86	30.09 ± 6.25	28.13 ± 6.76	30.51 ± 6.25	33.17 ± 6.60	**32.50 ± 4.75+**	26.84 ± 5.98	**28.23 ± 3.92-**	27.43 ± 5.89	26.29 ± 6.61	**25.72 ± 3.84-**
PL	10.85 ± 2.24	**10.71 ± 2.07-**	12.23 ± 2.55	11.20 ± 2.49	**12.22 ± 1.52+**	11.64 ± 2.14	11.57 ± 2.16	11.35 ± 2.51	11.73 ± 2.19	10.96 ± 2.05	10.73 ± 2.30	10.19 ± 2.25	10.09 ± 2.22	10.55 ± 2.79	9.93 ± 2.10	10.90 ± 2.78
ULL	10.56 ± 3.45	11.02 ± 2.80	10.69 ± 3.32	13.35 ± 3.09	13.11 ± 3.23	13.79 ± 3.00	12.74 ± 4.23	11.17 ± 4.26	14.17 ± 3.57	15.68 ± 6.04	**14.65 ± 3.07+**	**15.08 ± 4.86+**	**9.55 ± 2.45-**	11.41 ± 3.46	10.80 ± 3.00	13.29 ± 2.23
LLW	5.89 ± 0.82	**5.54 ± 0.68-**	6.27 ± 0.80	6.33 ± 0.81	6.06 ± 0.79	6.37 ± 0.96	6.24 ± 1.18	6.40 ± 1.34	6.27 ± 1.02	**7.09 ± 1.31+**	5.84 ± 0.98	6.26 ± 1.23	5.88 ± 0.68	5.86 ± 0.89	**5.14 ± 0.90-**	**5.59 ± 0.79-**
complex index	-2.33	-3.21	-2.45	-2.87	-3.76	-2.95	-2.46	-2.31	-2.36	-2.05	-2.46	-2.93	-2.49	-2.09	-1.91	-2.22
positive traits	1	3	4	2	8	3	4	3	2	3	6	3	2	0	1	1
negative traits	3	2	3	0	1	0	2	0	0	2	3	3	2	2	2	3

+ means this trait may have a significant positive effort for concerned traits, while - means this trait may have a significant negative effort for concerned traits.

The abbreviation of traits see **Table 2**.

The values in bold are the groups in which the maximum and minimum values have a statistically significant difference.

**Figure 3:**
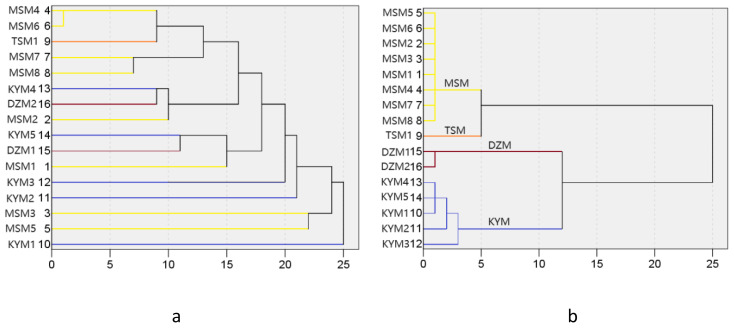
Cluster trees based on Euclidean distance of functional traits **(a)** and cluster trees based on geographical distance of provenances **(b)**. The color of each line represents different provenance. The serial number please see **Table 1**.

Based on Euclidean distance algorithm, we calculated the functional distance of each strain (**Table A1**). In order to investigate the relationship between functional traits and provenance environment, we calculated the relative geographic distance of provenance for each strain. The above distances are clustered and displayed in a tree graph (**Figure 3**). The cluster analysis was performed in SPSS 19.0 software. At the same time, principal component analysis (PCA, with Z-value centralization) was conducted for the trait values involved in the analysis to clarify the performance of each strain on different trait dimensions. PCA is performed in Canoco 4.5 software.

Based on PCA and Pearson correlation analysis, we discussed the relationship between the constructed key complex index and measurable traits in order to find out visual indicators that can be used for early identification of excellent strains. Since the key complex index were constructed based on Vt and BR, we did not analyze the correlation between these two traits and the key complex index. Correlation analysis was performed in STATISTICA 10.0. Based on the analysis results, we selected LCC and LW as the easy to measure traits, and then performing a linear fitting between them and key complex index. Linear fitting was performed in Oringin 8.0. Alsp, we used the hier.part package of R 3.4.5 software to decompose the key complex index and the functional traits involved in the analysis to reconfirm the robustness of the results.

## 3 Results

### 3.1 Excellent strains based on statistical screening of single functional traits

When considering only the functional advantages of a single functional trait, we found that strain No. 11 (optimal with 6 traits) and strain No. 5 (optimal with 5 traits) were significantly different from other strains (**Table 3**). Strain No. 11 had the highest BR, larger PL and LW, but the smallest Vt, Vb, RLA, and smaller LCC and LDMC. Strain No. 5 had the highest LA, LN, RLA, and its Vb, LW, and PL were also larger. It should be noted that strain No. 11 also had the highest GR (73.91%), while strain No. 5 had a very low GR (3.57%). Besides, we found no correlation between tannin and polyphenol content in different strains (**Figure 2**), indicating that the two are independent traits (Vasseur et al., 2012). Without considering the germination rate, strain No. 11 and strain No. 11 had opposite characteristics in many traits, but both had more favorable traits. If the germination rate is considered, strain No. 11 can be identified as an excellent strain, that is, it is characterized by high germination rate, strong disease resistance and stable functional traits.

### 3.2 Relationship between functional distance of strains and geographic distance of provenance

In general, the more geographically close individuals are, the more similar their functional traits may be, which is caused by two ecological processes: habitat filtering and dispersal limitation (Xu et al., 2017; Duan, 2023). However, in this study, it was found that cluster trees based on Euclidean distance of functional traits showed different patterns from cluster trees based on geographical distance of provenances (**Figure 3**). This indicates that the difference of provenance is not the reason for the formation of different character combinations in the strains. Therefore, environmental differences did not cause habitat filtering or environmental modification of the parents in the provenance area, and the reason for the formation of trait differentiation between strains was the genetic diversity of their parents. Therefore, the relationship between parental habitat and offspring traits was not discussed in subsequent analyses.

### 3.3 Combination of traits of different strains

**Figure 4:**
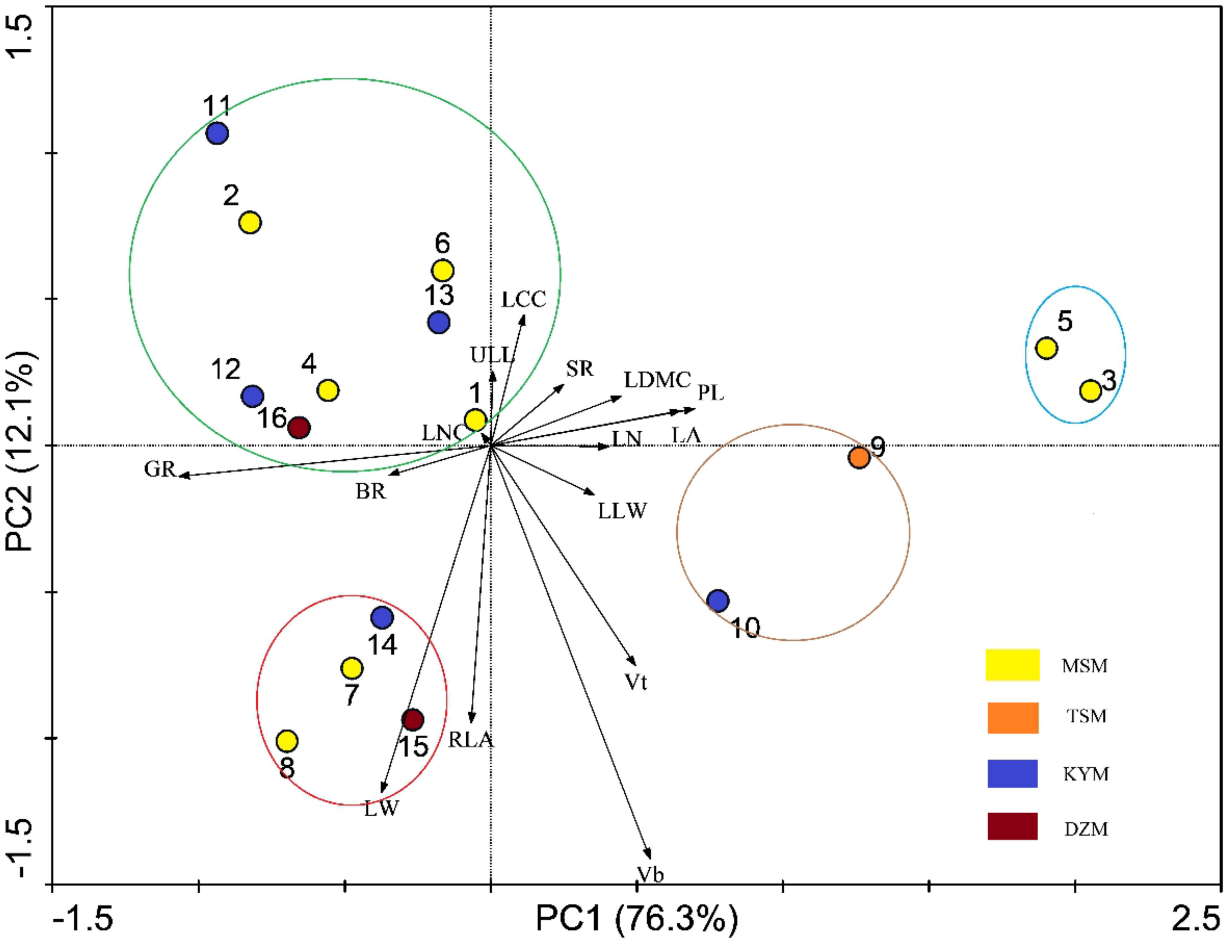
Principal component analysis of functional traits among different strains. The arrows’ included angle mean functional traits which correlated with PC1 and PC2, and the relationship between each other. Circles with number refer to the strains. The colored circles refer to different group of strains based on PCA. The abbreviation of traits see **Table 2**.

PCA analysis showed that the PCA1 and PCA2 axes could explain 88.4% of the trait variation of different strains participating in the analysis (**Figure 4**). PCA1 was positively correlated with leaf traits such as PL(correlation value=0.882), LA(correlation value=0.819), LN(correlation value=0.631), LDMC(correlation value=0.598), and negatively correlated with GR(correlation value=-0.910) and BR(correlation value=-0.499). PCA2 was positively correlated with LCC(correlation value=0.575), ULL(correlation value=0.418), and negatively correlated with Vb(correlation value=-0.913), Vt(correlation value=-0.522), RLA(correlation value=-0.588), and LW(correlation value=-0.615). The different strains were roughly clustered into four groups, and the four groups happened to fall into different quadrants. Vt and BR were selected to construct key complex index of *J. mandshurica*, and these two traits were significantly correlated with PCA axis as well. Therefore, we can select potential excellent strains in the third quadrant representing larger BR and larger Vb. In the third quadrant, the strains No. 15, 8, 7, and 14 were clustered together, indicating that they had similar trait combinations, that is, they may have higher LW and RLA but lower LN and LCC while having higher Vb and BR. In the first quadrant, where strains No. 3 and 5 are located, these traits were reversed with No. 15, 8, 7, 14. The fourth quadrant of strains No. 11, 2, 6, 4, 13, 12, 16, 1 represented higher BR, GR and LCC but lower Vt, Vb, LW, RLA and PL. The second quadrant, where Strains No. 9 and 10 were located, showed the opposite traits with strains located in fourth quadrant (**Figure 4**). We also found that the pattern of strains clustering in PCA was independent of geographical distance and functional distance. That is, strains with similar geographical distance or functional distance did not aggregate together during PCA analysis of traits (**Figures 3**, **4**).

### 3.4 Performance pattern and evaluation of key complex index

The key complex index we constructed showed that when focusing on growth amount and insect resistance, strain No. 15 was optimal, followed by strain No. 10, 14, 16 and 8. If the germination rate was taken into account, strain No. 10 was excluded (GR=14.39%, much lower than other strains). Compared with the excellent strains selected based on single functional traits, strain No. 5 and strain No. 11 both had low scores on key complex index. Moreover, strain No. 5 had the lowest score (**Table 3**). That is because strain No. 11 had the lowest Vt and strain No. 5 had the lowest BR, which was poor in the traits that received attention. Compared with the excellent strains screened by PCA results, No. 15, No. 14 and No. 8 were consistent. Among the 5 strains screened by the constructed key complex index, except No. 15 had the highest Vt, the other strains were not prominent in each trait, indicating that there were differences between the excellent strains screened by key complex index and the strains that screened by single functional traits.

**Figure 5:**
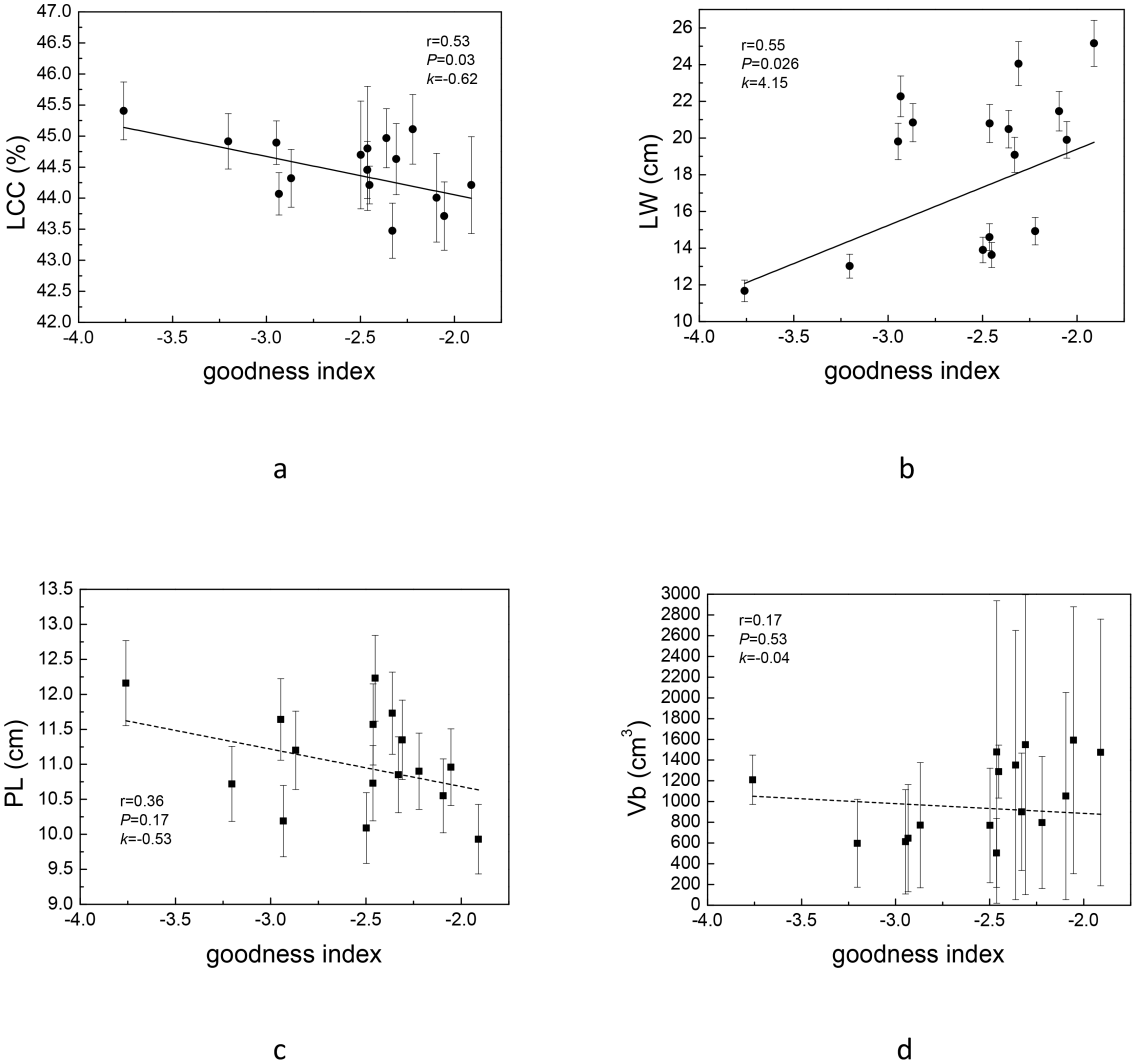
Linear regression of complex index (goodness index in graph) and leaf carbon content [LCC **(a)**], leaf width [LW **(b)**], petiole length [PL **(c)**] and annual branch volume [Vb **(d)**]. The solid line indicates that the regression is statistically significant, while dashed lines indicate that regression is not statistically significant.

In view of the large workload of key complex index calculation, we tried to select one or several easily measured traits associated with it to quickly screen strains. We found that, except Vt and BR, LCC and LW were correlated with the newly constructed complex index (**Table 3**). PCA analysis also showed that Vt and BR used to construct complex index were orthogonal (**Figure 4**), indicating that the two pairs of traits were independent of each other, and the traits on the bisector line of the angle between the two pairs of traits could be selected as the “proxy” of the complex index. Referring to **Figure 4**, we can still see that LCC and LW meet this setting. Subsequent variance decomposition showed that Vb (19.14%), LCC (14.45%), PL (12.15%) and LW (11.19%) accounted for the most variation of complex index, which proved that the constructed complex index had the possibility of visualization through measurable traits from above three aspects. Linear regression showed that the constructed key complex index tended to decrease with the increase of LCC (**Figure 5a**) and increase with the increase of LW, and the slope (k-value) was larger on the LW gradient (**Figure 5b**). However, the key complex index were not linearly related to Vb and PL (**Figures 5c, d**). Therefore, for the key traits of *J. mandshurica*, the goodness of key complex index can be quickly identified by the size of LCC and LW.

## 4 Discussion

### 4.1 The key complex index provides a new perspective for strain selection

In the past, when selection of strains, the performance of different strains was often discussed separately on different single traits. This method can give different optimal solutions of strains in multiple application scenarios. However, different traits may have similar ecological effects, but their performance patterns may be opposite, which brings problems for managers to make selection decisions. For example, Wright et al. (2004) have shown that there is a trade-off between leaf nitrogen content and traits such as leaf lifespan and specific leaf area, although the improvement of these traits is beneficial for plants in different ways. The ability of plants to acquire resources is limited, while increasing the investment in some traits, they will inevitably reduce the investment in some traits, and sacrifice some dominant traits to maintain the prominence of other traits, so as to avoid overlapping with other species or the same individual in the competition of ecological niche (Anderegg et al., 2018). Therefore, only discussing the advantages of a single strain on a single trait may cause confusion in the conclusion. We propose that for different application scenarios of forests, researchers should establish key complex index which are composed of the traits of concern to evaluate the availability of the strains in this scenario. In this study, the *J. mandshurica* plantation will be used for landscaping, so we chose the indicators of growth amount (replaced by Vt) and insect resistance (combined indicator of PC and TC) that managers are most concerned about to construct this key complex index. Compared with the single trait selection, the complex index selection had significant difference. For example, when using a single trait for evaluation, the candidate strains No. 5 and No. 11 had their own outstanding advantages in many trait dimensions, but for the traits that the managers paid attention to, Vt and BR, the two strains each showed two opposite performance patterns, leading to the elimination of advantages when using complex index for screening. By observing the traits of the two strains No. 5 and No. 11, we can find that in many traits, the two lines showed opposite patterns. The results showed that No. 5 and No. 11 strains adopted two opposite strategies in trait combination (Anderegg et al., 2018). For example, strain No. 5 may have preferred a strategy of high photosynthetic capacity and rapid growth (as evidenced by its high current shoot volume, high LNC, and large lenticel area (indicating the laxity of its pericarp), but at the expense of reducing secondary metabolite synthesis and accumulation (having the lowest polyphenol and tannin content). Strain No. 11 preferred slow growth (as evidenced by its lowest Vt, Vb, RLA and LNC) but invested resources in the synthesis and accumulation of secondary metabolites (its tannin content was the highest among the strains), thus establishing its competitive advantage in different aspects. However, these advantages are of little value to garden managers. On the contrary, several strains screened by key complex index had no advantage in single traits but were very balanced in growth amount and insect resistance. The PCA results also showed that growth amount and insect resistance are orthogonal independent traits, again proving that the two sets of traits are not covariant but are trade-offs. Therefore, it is unlikely that the strains screened by complex index will achieve high scores on both sets of traits, but it must be very balanced. Therefore, the construction of complex does provide a newer perspective of strain selection compared to using a single trait.

It should be pointed out that when constructing complex index, it is also necessary to pay attention to the robustness of traits. Some traits are highly affected by the environment and show strong ability of environmental modification. For example, the LA of the same species of plant can show high variability depending on the habitat in which it is located (Wang et al., 2019). Although this experiment was carried out in a homogeneous garden, the trait variation caused by environmental factors can be avoided to the greatest extent, but if highly variable traits are selected to construct complex index, its application scope will be greatly limited. The conclusions drawn in this way can only be applied in the local area of the experiment, and it is difficult to generalize about other areas with different environments. Therefore, in the selection of traits, it is necessary to explore whether the production of these traits is influenced by ecological processes such as environmental filtering (resulting in different traits in different habitats) or dispersal limitation (similar phylogenetic relationships in similar habitats, Wang et al., 2018; Whitman and Aarssen, 2010). Before the analysis, this study first eliminated the interference of different ecological processes on the traits by comparing the trait distance clustering tree and the geographical distance clustering tree, and proved that the trait performance of the strains was based on its genetic characteristics. Most of the progeny of a single tree of *J. mandshurica* were formed by the mating of 1-2 nearby trees, which also ensured the stability of the strain traits (Bai et al., 2007). However, it is also necessary to avoid using highly phylogenetically conserved traits to construct complex index, such as flower color, seed mass (For large seeds such as *J. mandshurica*, the variation of seed mass is large, which can be used to construct complex index, Li et al., 2022), etc. Even if these traits are of concern to managers in some scenarios, given their low intraspecific variability, it is not appropriate to participate in the construction of complex index. Besides, researchers can set the weight of each functional trait to construct complex index (Chen et al., 2023), especially when their significance for manager is different.

### 4.2 Easily measurable traits can be applied to the rapid screening of excellent strains

The correlation between traits has long been an area of concern for ecologists. The traits of different dimensions reflect the adaptability of plants to different aspects, so there may be three modes of covariation, orthogonal and tradeoff among traits (Liu et al., 2019; Zuleta et al., 2022). The key complex index we constructed have been proved to be suitable for the selection of excellent trait combinations that attract the attention of managers, but the construction of complex index often requires the determination of many trait values, which is time-consuming and laborious. Therefore, how to visualize the complex index, so as to realize the early and rapid screening of excellent strains, is a challenge for managers in the application of complex index. Therefore, based on the correlation of traits, we tried to find the measurable traits that were covariant with the complex index as the “agents”. In this study, we found that LW and LCC can be used as visual indicators of complex index. For plants, the increase of LCC means the increase of carbon-containing organic matter in leaves, including structural carbon (cellulose, lignin) and non-structural carbon (soluble sugar, starch, etc., as photosynthates; and phenolic substances, flavonoids, etc., as secondary metabolites). In fact, we found that with the increase of LCC, complex index showed a pattern of decline. LW has no obvious ecological function, and its value does not represent ecological significance. In this study, however, it was found that LW could represent the complex traits concerned by managers. On the one hand, the association between traits may be due to their genetic linkage (for example, the same or more genes regulate both traits at the same time), on the other hand, the association may be “coincidental”, that is, not causative. Therefore, for measurable traits that can characterize complex index, subsequent researchers should distinguish between the two cases. If it is established that there is a genetic link between these traits, the correlation of these traits may be applicable in any scenario of the same species; If the two traits are not related at the phylogenetic level, the correlation between the two pairs of traits will be non-causal. Such a situation would limit the selection of excellent strains through measurable traits. For this study, the covariation of LCC with complex index may be more robust. Although the increase of LCC may lead to the increase of secondary metabolites or the accumulation of structural carbon, and thus to the improvement of complex index, there is another possibility that the increase of LCC is mostly due to structural carbon, and the polyphenols and tannins allocated to leaves are not increased, but decreased. This was confirmed by our trait association analysis (**Figure 2**). The correlation between traits with obvious ecological trade-off attributes should have a certain genetic basis. Therefore, the relationship between LCC and the complex index may not be accidental. We can use LCC, an easily measured trait, to characterize this complex index. Still, it should be noted that the above analysis is necessary if the case is to be generalized to other species. Different species and different managers pay attention to different traits, and the proxy measurable traits selected should be different.

## 5 Conclusion

Compared with the previous method of multiple screening on a single trait, we focused on the growth amount and insect resistance indicators that managers are concerned about, constructed a key complex goodness index, and demonstrated the usability of this complex index. The study showed that the excellent strains (No. 15 from Dazeshan) based on complex index screening are different from those based on single trait screening (No.5 from Mengshan and No. 11 from Kunyushan). They may not have the best performance in single trait dimension, but they have more balanced trait values in the combination of traits weighing each other. We suggest that the functional traits involved in constructing key complex index should be non-highly variable but not highly phylogenetically conserved. Managers should choose different traits and construct different key complex index for different concerns. We also tried to find a means of visualizing complex traits by selecting one or more easily measurable traits that have stable ecological associations with complex index as proxies. Based on the *J. mandshurica*, measurable traits (LCC) can be found that are ecologically associated with the constructed complex index, but for other species or other traits of concern, re-analysis is needed. In conclusion, the construction of key complex index based on the needs of managers provides a new perspective for the optimization of strains.

## Data Availability

The original contributions presented in the study are included in the article/supplementary material. Further inquiries can be directed to the corresponding author.

## Appendix

**Table A1:** Euclidean distance of functional traits between stains.

Stain code	Euclidean distance															
	1	2	3	4	5	6	7	8	9	10	11	12	13	14	15	16
1	.000	319.817	394.400	153.380	367.570	320.256	582.761	651.232	455.843	709.911	404.343	294.917	144.747	176.353	580.602	161.369
2	319.817	.000	707.501	219.960	672.557	208.383	890.053	953.671	764.072	1025.316	130.342	86.193	184.677	458.227	878.641	204.740
3	394.400	707.501	.000	522.371	172.133	684.949	204.080	276.867	83.181	328.773	792.541	669.950	527.478	267.476	225.557	519.356
4	153.380	219.960	522.371	.000	464.254	177.864	706.636	779.626	583.811	829.503	276.324	219.298	88.608	309.653	715.260	166.911
5	367.570	672.557	172.133	464.254	.000	603.856	311.970	402.846	229.840	384.608	736.875	652.043	492.263	307.772	378.702	512.339
6	320.256	208.383	684.949	177.864	603.856	.000	870.492	947.704	749.445	981.454	174.660	257.434	222.264	485.105	886.760	302.003
7	582.761	890.053	204.080	706.636	311.970	870.492	.000	97.266	139.246	177.352	975.483	849.641	712.772	441.664	129.378	698.384
8	651.232	953.671	276.867	779.626	402.846	947.704	97.266	.000	209.372	199.298	1044.963	908.780	780.112	499.220	103.535	757.250
9	455.843	764.072	83.181	583.811	229.840	749.445	139.246	209.372	.000	285.724	851.772	722.440	585.872	315.624	160.538	571.908
10	709.911	1025.316	328.773	829.503	384.608	981.454	177.352	199.298	285.724	.000	1102.056	991.392	844.678	588.841	271.206	841.333
11	404.343	130.342	792.541	276.324	736.875	174.660	975.483	1044.963	851.772	1102.056	.000	197.573	274.536	557.507	975.493	318.403
12	294.917	86.193	669.950	219.298	652.043	257.434	849.641	908.780	722.440	991.392	197.573	.000	171.590	415.393	831.090	163.898
13	144.747	184.677	527.478	88.608	492.263	222.264	712.772	780.112	585.872	844.678	274.536	171.590	.000	288.699	707.500	90.445
14	176.353	458.227	267.476	309.653	307.772	485.105	441.664	499.220	315.624	588.841	557.507	415.393	288.699	.000	421.686	260.422
15	580.602	878.641	225.557	715.260	378.702	886.760	129.378	103.535	160.538	271.206	975.493	831.090	707.500	421.686	.000	678.688
16	161.369	204.740	519.356	166.911	512.339	302.003	698.384	757.250	571.908	841.333	318.403	163.898	90.445	260.422	678.688	.000
